# Transcriptomic analyses and experimental verification reveal potential biomarkers and biological pathways of urinary tract infection

**DOI:** 10.1080/21655979.2021.1987081

**Published:** 2021-10-21

**Authors:** Wenbo Yang, Peng Liu, Yuling Zheng, Zhongtian Wang, Wenhua Huang, Hua jiang, Qingyu Lv, Yuhao Ren, Yongqiang Jiang, Liping Sun

**Affiliations:** a Changchun University of Chinese Medicine, Changchun, Jilin,China; b State Key Laboratory of Pathogen and Biosecurity, Institute of Microbiology and Epidemiology, Academy of Military Medical Sciences, Beijing, China; c Affiliated Hospital of Changchun University of Chinese Medicine, Changchun, Jilin, China

**Keywords:** Differentially expressed gene, urinary tract infection, protein-protein interaction network, tissue-specific gene expression

## Abstract

Urinary tract infection (UTI) is a common infectious disease. Urinary tract pathogenic Escherichia coli (UPEC) is the main cause of UTIs. At present, antibiotics are mainly used for the treatment of UTIs. However, with the increase of drug resistance, the course of the disease is prolonged. Therefore, identifying the receptors and signal pathways of host cells and tissues will further our understanding of the pathogenesis of UTIs and help in the development of new drug treatments. We used two public microarray datasets (GSE43790, GSE124917) in the Gene Expression Omnibus (GEO) database to identify differentially expressed genes (DEGs) between UTI and normal cell samples. A functional analysis based on Gene Ontology (GO) data, a pathway enrichment analysis based on Kyoto Encyclopedia of Genes and Genomes (KEGG) data and a protein-protein interaction analysis identified the main potential biomarkers and verified them in animal tissues. A total of 147 up-regulated genes and 40 down-regulated genes were identified. GO enrichment analysis showed that these functional changes relate to the terms response to lipopolysaccharide, regulation of cytokine production, and regulation of the inflammatory response. KEGG analysis indicated that urinary tract infections likely involve the TNF-αsignaling pathways. The 20 hub genes were selected from the protein-protein interaction network, and the highly significant hub genes were verified by animal experiments. Our findings provide potential targets for exploring new treatments for urinary tract infections. After a comprehensive analysis of the GEO database, these results may facilitate development of new diagnosis and treatment strategies for urinary tract infections.

## Introduction

Urinary tract infection is one of the most common infectious diseases worldwide. Including children and adults, about 150 million people worldwide suffer from UTIs every year [1]. Urinary tract infections are mainly caused by bacteria. Urinary tract pathogenic Escherichia coli (UPEC) is the most common pathogen. 75% of uncomplicated UTIs and 65% of complicated UTIs are caused by UPEC [2]. UTIs have caused a heavy economic burden on the public and society. Although antibiotics are currently recommended to treat UTIs, the increase in drug resistance affects the therapeutic effect of these drugs on UTIs [3]. The main virulence factors of UPEC causing UTIs are its fimbriae, hemolysin, and iron acquisition system [4]. The pathogenic mechanism of UPEC is the main research direction of UTIs, while research on the inflammatory response in humans have received less attention. Therefore, elucidating the specific pathogenesis of UTIs in the human body and exploring potential biomarkers are helpful to the development of new treatment methods.

Microarray and bioinformatics analyses provide new ideas for exploring the molecular mechanisms of diseases [5]. They have also changed people’s understanding of disease diagnosis and treatment. The microarray dataset can analyze the key genes and pathways involved in UTIs. However, a single microarray dataset cannot fully reflect the disease process. At the same time, different microarray datasets may also produce diverse results in differential expression. Therefore, we conducted a comprehensive analysis at the transcription level of the expression differences, molecular interactions and biological functions of UTIs.

Two mRNA microarray datasets obtained from the GEO database were used to analyze the differences between UPEC-infected kidney cell samples and uninfected (normal) samples. In addition, GO function and KEGG enrichment analyses, protein-protein interaction analysis, and determination of the molecular mechanism of UTI from the receptor perspective were also carried out and verified in animal tissues.

Therefore, this study aims to evaluate the key biomarkers of urinary tract infection through bioinformatics methods and experimental verification. Then an important gene network of UTI that may be closely related to inflammation was established, and a meaningful framework was provided for exploring the key biomarkers and pathways related to UTI diagnosis and treatment.

## Materials and methods

### Gene expression omnibus datasets

**Figure 1.**
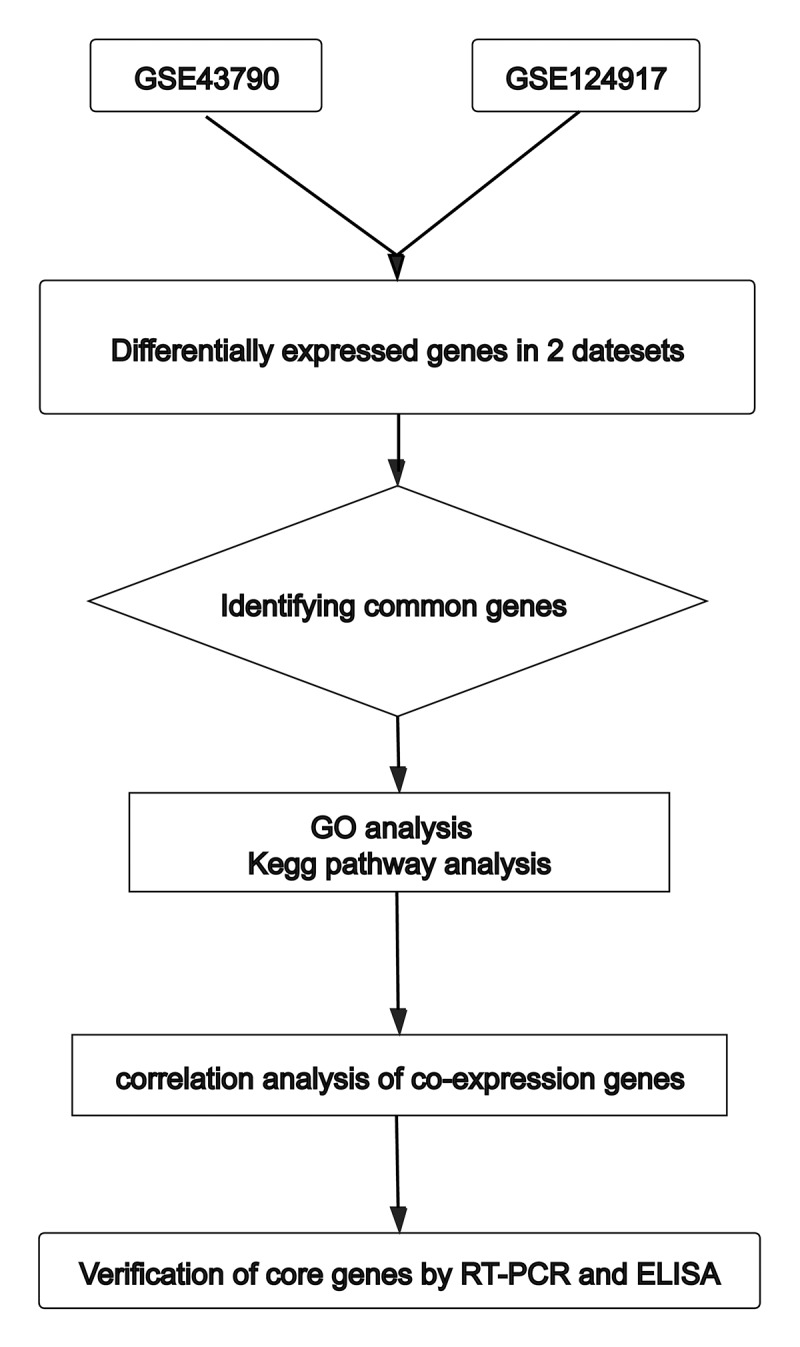
Flow chart of analyses performed in this research

The transcriptome profile datasets were obtained from the NCBI GEO databases [6] (http://www.ncbi.nlm.nih.gov/geo/). We searched using the keyword ‘urinary tract infection’ and two data sets, GSE43790 and GSE124917, were selected. GSE43790 is derived from GPL6947 (Illumina HumanHT-12 V3.0 expression beadchip) and has three normal samples and five samples infected by UPEC [7]. GSE124917 is derived from GPL21185 (Agilent-072363 SurePrint G3 Human GE v3 8x60K Microarray), and it has three normal samples and three samples infected with UPEC [8]. Each sample’s data used in this section were all downloaded from GEO database; therefore, no patient consent or ethics committee approval was necessary. Our research design flow chart is shown in Figure 1.

### Identification of DEGs

The online difference analysis tool GEO2R [9] was used to find DEGs shared between the normal and UPEC-infected samples. Those that met the standard adjusted P < 0.05 and |logFC|>2.0 were defined as DEGs. We used the online tool Bioinformatics (http://www.bioinformatics.com.cn/) to create our figures.

### KEGG and GO enrichment analyses of DEGs

Metascape, an online enrichment analysis tool, was used to perform an enrichment analysis at the molecular and functional levels [10]. We used metascape to perform the GO and KEGG enrichment analyses on the DEGs. Gene Ontology analysis categorizes the functions of DEGs into three domains: biological process (BP), molecular function (MF), and cellular component (CC). KEGG indicates the main pathways in which the DEGs may participate. P < 0.01, a minimum count of 3, and an enrichment factor > 1.5 defined by metascape was considered statistically significant.

### Protein-protein interaction (PPI) network construction and hub gene identification

STRING is an online biological database for predicting the interactions between proteins and proteins [11]. Cytoscape 3.7.2 is a software that visualizes the PPI network [12], and the Cytoscape plug-in Cytohubba [13] can be used to select hub genes. In this study, the STRING database was used for the construction of the PPI network, and median confidence scores >0.9 were considered statistically significant. Then we used Cytoscape to visually analyze our PPI network. Cytohubba was used to find the hub genes in the DEGs and sort them according to the Maximal clique centrality (MCC) method. The top 20 genes are considered hub genes by MCC method.

### Tissue specimens, Real Time-PCR, and ELISA analysis

RT-PCR was used to analyze the expression levels of hub genes. Bacterial strain UPEC307 was isolated from a 56-year-old female patient with acute pyelonephritis. In LB medium, we cultured UPEC307 overnight placed on a shaker at 180 r/min and at 37°C. Then 50 ul of the bacterial solution was transferred to 5 ml of fresh LB medium and cultured for 3 hours to reach the mid-log phase. The mid-log phase culture was centrifuged at 8000 r/min for 3 min to collect the bacteria and remove the supernatant.

C57BL/6 mice were purchased from Charles River Laboratories China (Beijing, China). The animal care and use procedures have been approved by the Institutional Animal Care and Use Committee of the Academy of Military Medical Science (AMMS, Beijing, China).the ethics committee, and all applicable institutional and government regulations regarding the ethical use of animals have been complied with. Mice were anesthetized and inoculated via the urethra with 100 ul UPEC307 resuspended in PBS. They were sacrificed at 6 h after infection, and their kidneys were taken aseptically. Then the mouse kidneys were weighed, PBS was added and the kidneys were crushed and ground, centrifuged at 4°C, 10,000 × g, 10 min, and the supernatant was taken.

The RNeasy Mini Kit (Qiagen, Hilden, Germany) was used to extract total RNA from cells, and then the RNA was transcribed into cDNA using the MightyScript First Strand cDNA Synthesis Master Mix (Sangon Biotech, Shanghai, China). We used SYBR@Green Master Mix (Applied Biosystems) for RT-PCR, and the housekeeping gene GAPDH served as an internal control. The PCR primers used in this study are shown in Table 1.The TNF -α, IL-1β, IL-6 ELISA Kit were obtained from Neobioscience Technology (Shenzhen, China).

**Table 1.** Primers used in this study

Gene	Forward Primer	Reverse Primer
TNF	TCCAGGCGGTGCCTATGT	GCCCCTGCCACAAGCA
IL-6	CCACGGCCTTCCCTACTTC	TTGGGAGTGGTATCCTCTGTGA
IL-1β	AGTTGACGGACCCCAAAAGA	GGACAGCCCAGGTCAAAGG
GAPDH	CATGGCCTTCCGTGTTCCTA	GCGGCACGTCAGATCCA

## Results

This study used bioinformatics analysis and experimental verification methods to reveal potential biomarkers of urinary tract infection from transcriptomics. We screened out the main up-regulated and down-regulated genes of urinary tract infection, and then obtained the main biological processes and pathways involved in urinary tract infection, and then verified it through animal experiments. Finally, it provides a reliable target for the diagnosis and treatment of urinary tract infections.

### Identification of DEGs

**Figure 2.**
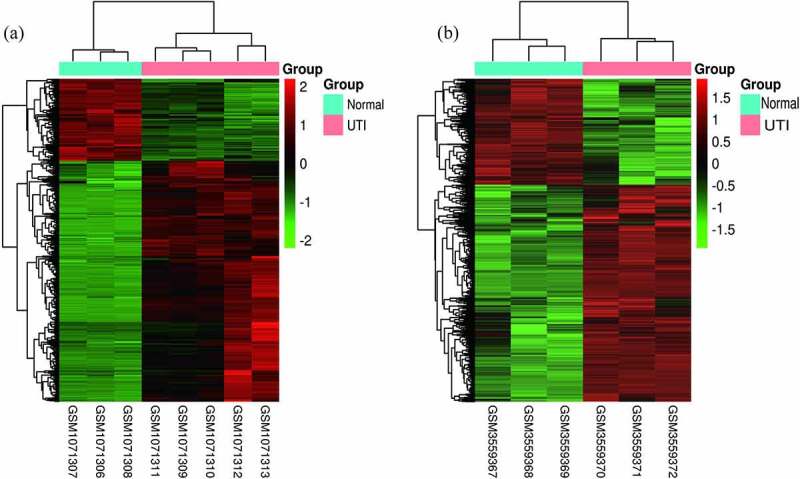
Heat maps of potential DEGs in the two microarray datasets (a) GSE43790 and (b) GSE124917

**Figure 3.**
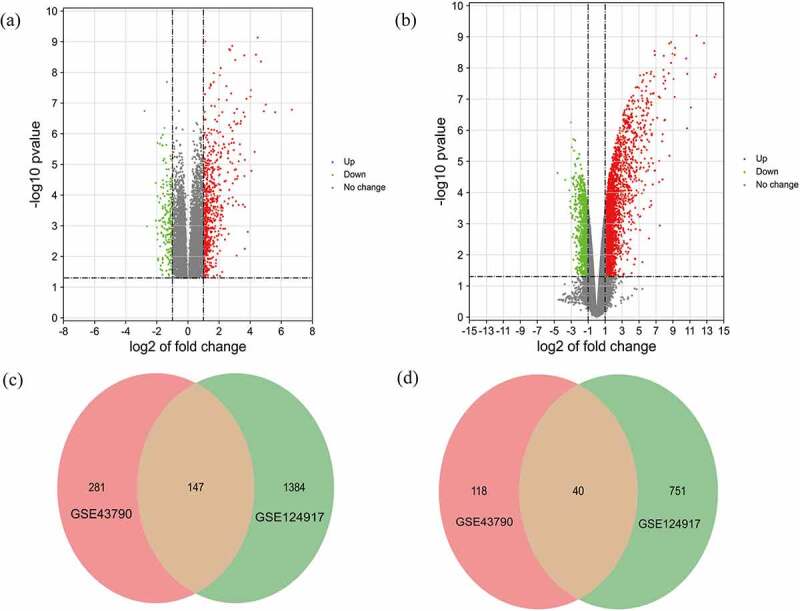
Volcano maps of the distributions of all DEGs in (a) GSE43790 and (b) GSE124917. Red, green, and gray colors respectively represent up-regulated genes, down-regulated genes, and genes with no difference in expression. Venn diagrams showing (c) 147 up-regulated DEGs and (d) 40 down-regulated genes common to both GSE43790 and GSE124917

The criteria to identify DEGs, P < 0.05 and |logFC|>2.0, produced a total of 428 up-regulated genes and 158 down-regulated genes for the GSE43790 dataset and a total of 1531 up-regulated genes and 791 down-regulated genes for the GSE124917 dataset. Figures 2a and 2b show heat maps distinguishing mRNA expression between UTI samples and normal samples. Additionally, these DEGs were also visualized by a volcano map. The two microarray datasets shared totals of 147 up-regulated genes and 40 down-regulated genes (Figure 3).

### KEGG and GO enrichment analyses of DEGs

**Figure 4.**
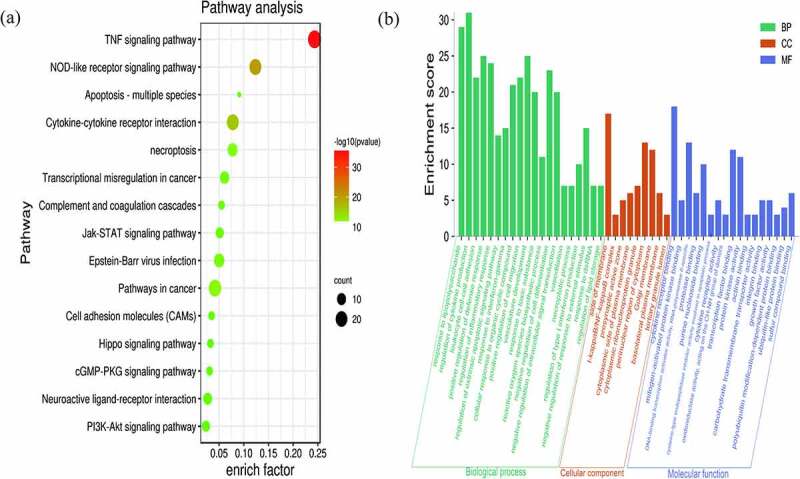
Significant KEGG pathways (a) and GO terms (b) enriched with DEGs. The size of the bubble indicates the enrichment score, and the color indicates the significance of enrichment

Metascape was used for a GO function analysis and KEGG pathway enrichment analysis of DEGs. According to the enrichment results, a total of 20 BP, 9 CC, and 17 MF terms were obtained based on GO and 15 pathways were obtained based on KEGG (Figure 4). And the top five terms of GO function analysis and KEGG pathway are presented (Table 2).

**Table 2.** GO and KEGG pathway enrichment analysis of the most important items in hub genes

Category	Pathway ID	Pathway description	Count	P value
GO-BP	GO:0032496	response to lipopolysaccharide	29	2.95121E-24
GO-BP	GO:0001817	regulation of cytokine production	31	1.69824E-15
GO-BP	GO:0007159	leukocyte cell-cell adhesion	22	5.24807E-15
GO-BP	GO:0031349	positive regulation of defense response	25	1.99526E-14
GO-BP	GO:0050727	regulation of inflammatory response	24	1.25893E-13
GO-CC	GO:0098552	side of membrane	17	7.58578E-07
GO-CC	GO:0033256	I-kappaB/NF-kappaB complex	3	1.12202E-06
GO-CC	GO:0048786	presynaptic active zone	5	9.54993E-05
GO-CC	GO:0009898	cytoplasmic side of plasma membrane	6	0.000870964
GO-CC	GO:0036464	cytoplasmic ribonucleoprotein granule	7	0.001
GO-MF	GO:0005126	cytokine receptor binding	18	3.01995E-13
GO-MF	GO:0051019	mitogen-activated protein kinase binding	5	2.81838E-06
GO-MF	GO:0001228	DNA-binding transcription activator activity,RNA polymerase II-specific	13	9.12011E-06
GO-MF	GO:0002020	protease binding	6	0.000245471
GO-MF	GO:0001883	purine nucleoside binding	10	0.00025704
KEGG-PATHWAY	hsa04668	TNF signaling pathway	28	3.16228E-36
KEGG-PATHWAY	ko04621	NOD-like receptor signaling pathway	21	8.51138E-21
KEGG-PATHWAY	ko04060	Cytokine-cytokine receptor interaction	21	1.28825E-16
KEGG-PATHWAY	hsa05200	Pathways in cancer	24	8.51138E-13
KEGG-PATHWAY	hsa04217	necroptosis	13	1.04713E-10

**Figure 5.**
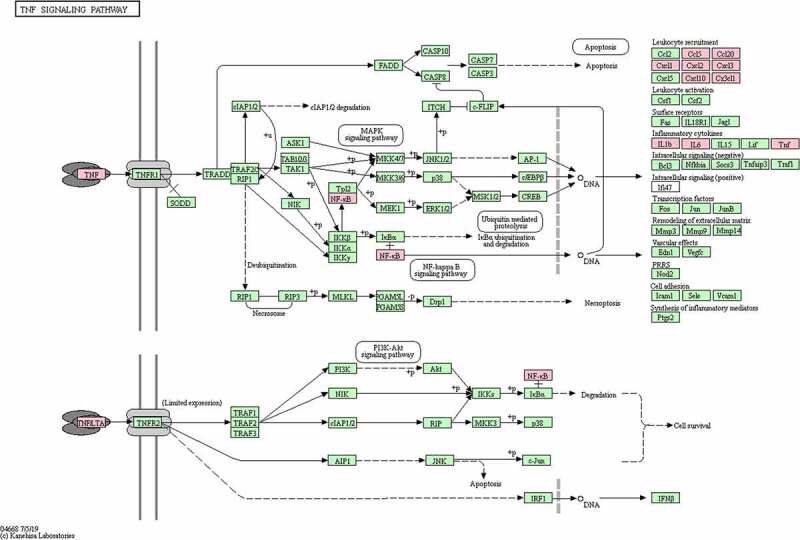
Hub genes in the TNF-α signaling pathway

In the GO analysis, DEGs that categorized in the BP ontology mainly annotated to the terms response to lipopolysaccharide, regulation of cytokine production, leukocyte cell-cell adhesion, positive regulation of defense response, and regulation of inflammatory response. The CC annotated terms primarily consisted of side of membrane, I-kappaB/NF-kappaB complex, presynaptic active zone, cytoplasmic side of plasma membrane, and cytoplasmic ribonucleoprotein granule. The MF terms were cytokine receptor binding, mitogen-activated protein kinase binding, DNA-binding transcription activator activity, RNA polymerase II-specific, protease binding, and pure nucleoside binding (Figure 4a). The main KEGG pathways were TNF signaling pathway, NOD-like receptor signaling pathway, Cytokine-cytokine receptor interaction, Pathways in cancer, and necroptosis (Figure 4b). Among them, TNF signaling pathway was the most significant pathway of enrichment (Figure 5).

### PPI network construction and hub gene identification

**Figure 6.**
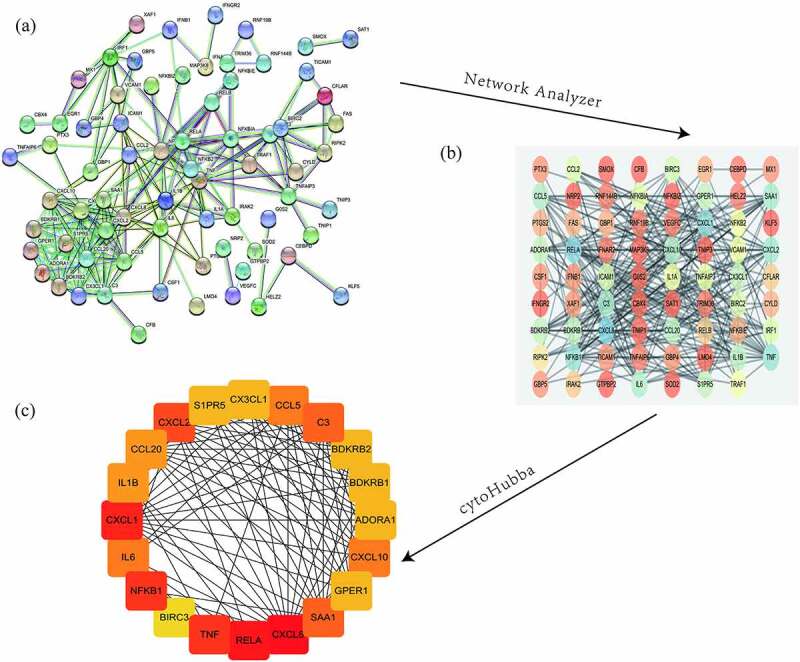
Protein-protein interaction analysis and identification of hub genes. The STRING database-predicted interactions of the 187 up- and down-regulated DEGs shared between the two microarray datasets (a). The Cytoscape plug-in Network Analyzer was applied to analyze the data (b), followed by the Cytohubba plug-in to analyze hub genes to obtain the highest ranking genes (top 20 shown)

In order to further explore the biological roles of the identified DEGs, the STRING database was used to construct a PPI network. Then we analyzed the network using Cytoscape and its tools, NetworkAnalyzer and the cytoHubba plugin, and presented results in Figure 6. The 20 genes with the highest scores were defined as hub genes. These genes are CXCL8, RELA, CXCL1, TNF, NFKB1, CXCL2, C3, SAA1, CCL5, CXCL10, IL6, CCL20, IL1B, BDKRB2, GPER1, S1PR5, BDKRB1, CX3CL1, and ADORA1 have significant differences in the two databases (Table 3). These differential genes may play an important role in the occurrence and development of urinary tract infections.

**Table 3.** The most significant upregulated and downregulated genes in DEGs

**Gene symbol**	**Gene description**	**logFC**		**P value**		**Regulation**
		GSE43790	GSE124917	GSE43790	GSE124917	
CXCL8	Interleukin-8	4.37	5.37	2.63E-09	9.54E-08	Upregulated
RELA	Transcription factor p65	1.27	2.25	7.33E-04	7.27E-06	Upregulated
CXCL1	Growth-regulated alpha protein	4.88	5.73	1.85E-07	5.73E-05	Upregulated
TNF	Tumor necrosis factor	3.34	4.56	2.20E-06	1.37E-02	Upregulated
NFKB1	Nuclear factor NF-kappa-B p105 subunit	1.53	2.01	4.27E-04	8.94E-06	Upregulated
CXCL2	C-X-C motif chemokine 2	3.76	5.98	7.24E-08	2.01E-06	Upregulated
C3	Complement C3	1.18	4.30	8.46E-03	1.45E-04	Upregulated
SAA1	Serum amyloid A-1 protein	1.07	4.13	4.91E-03	3.86E-02	Upregulated
CCL5	C-C motif chemokine 5	1.39	10.65	1.60E-02	8.65E-07	Upregulated
CXCL10	C-X-C motif chemokine 10	3.66	14.04	7.13E-06	1.58E-08	Upregulated
IL6	Interleukin-6	4.01	3.79	6.15E-08	3.07E-05	Upregulated
CCL20	C-C motif chemokine 20	5.61	7.37	1.99E-07	4.61E-05	Upregulated
IL1B	Interleukin-1 beta	2.87	6.27	1.19E-04	2.93E-04	Upregulated
BDKRB2	B2 bradykinin receptor	1.93	1.43	1.47E-05	3.41E-02	Upregulated
GPER1	G-protein coupled estrogen receptor 1	−1.62	−1.41	1.12E-02	3.57E-04	Downregulated
S1PR5	Sphingosine 1-phosphate receptor 5	−1.78	−1.98	6.58E-04	3.26E-02	Downregulated
BDKRB1	B1 bradykinin receptor	3.09	3.53	1.45E-05	2.35E-05	Upregulated
CX_3_CL1	Fractalkine	3.63	6.18	2.39E-05	2.34E-06	Upregulated
BIRC3	Baculoviral IAP repeat-containing protein 3	3.04	5.29	3.96E-09	5.30E-06	Upregulated
ADORA1	Adenosine receptor A1	−1.02	−1.06	9.53E-03	1.13E-02	Downregulated

### Verification of potential biomarker expression by Real Time-qPCR, and ELISA analysis

**Figure 7.**
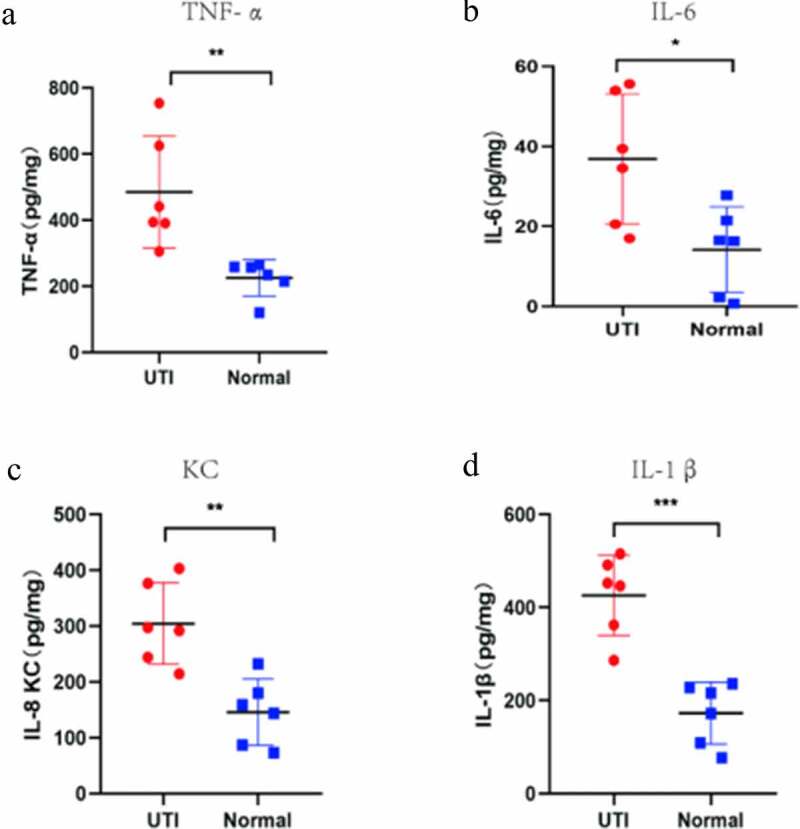
ELISA-detected expression of four hub genes in six un-infected kidneys and six UPEC-infected kidneys in mice. *P < 0.05, **P < 0.01, using unpaired T-test

**Figure 8.**
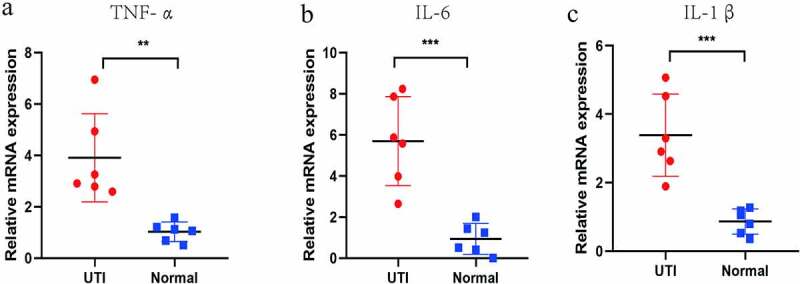
RT-PCR-detected differential expression of IL-β, IL-6 and TNF-α in six un-infected kidneys and six UPEC-infected kidneys in mice. **P < 0.01, ***P < 0.001 using unpaired T-test

The hub genes were verified by RT-PCR and ELISA methods. The expression of TNF-α, IL1β, IL-6 and KC (only detected by ELISA) were detected (Figures 7, 8). Compared with normal group, the TNF-α, IL-1β, IL-6 and KC genes in the mouse kidney tissues infected by UPEC were significantly up-regulated, which is consistent with the above bioinformatics analysis results.

## Discussion

Urinary tract infection is one of the common public health problems endangering human health. In clinical practice, UTI is mainly caused by bacterial infection [14], especially urinary tract pathogenic Escherichia coli (UPEC). An estimated 75% of uncomplicated UTIs and 65% complicated UTIs are caused by UPEC [2]. In the pathogenesis of urinary tract infection, the adhesion and invasion of UPEC are the main factors [4]. Antibiotics are currently the main method for the treatment of bacterial urinary tract infections [1], but due to the increase in antibiotic resistance, the recurrence rate of UTI is high, and the treatment period is prolonged, which has caused serious medical burdens [15,16]. The current research on UTI is mainly on how to reduce the colonization of bacteria in the human body, but there is very little research on the inflammatory response in the human body during the infection process. The rapid development and application of microarray technology has revealed genetic changes in the human body during infection, which may provide new targets in the search for new drugs to treat UTI.

This study uses bioinformatics methods to explore the biomarkers and hub genes in the signaling pathway of UTI. Here, we analyzed two microarray datasets to obtain DEGs and hub genes of UTI. A total of 187 shared DEGs (147 co-upregulated and 40 co-downregulated genes) were identified in the two datasets. Then GO function and KEGG pathway enrichment analyses were performed with the DEGs, a PPI network was constructed, and the hub genes in the identified KEGG pathways were verified in animal experiments.

Inflammation is not an independent pathogenic factor of UTI, nor can it explain all the physiological and pathological mechanisms in the pathogenesis of UTI. However, inflammation does play an important role in the pathogenesis of UTI. Our PPI network illustrates the potential relationships between the DEGs and 20 hub genes. Except for GPER1, S1PR5, BDKRB1, and ADORA1, all other genes were down-regulated. IL-6 is a biomarker with high sensitivity and specificity for UTI; the biomarker is related to the severity of UTI [17]. Both IL6 and IL-8 are activated and released in the immune response to UTI [8], and both are related to the severity of UTI [17]. Unfortunately, neither marker can be used to distinguish acute pyelonephritis from acute cystitis [18],which is verified in the urine and blood of children. The pro-inflammatory cytokines TNF-α, IL-8, IL-6, and IL1B can significantly promote the growth of UPEC [19,20].The colonization of UPEC can also activate RELA and NFKB1, and Inhibition of NF-KB can lead to long-term colonization of UPEC in the bladder [21]. Moreover, the activation of IL-6 and NFKB is mediated by TLR4 [22]. Consequently, IL-6 can induce the expression of CX3CL1 [23]. Some studies have demonstrated that inhibiting NFKB can reduce UTIs [24]. On the contrary, inhibiting NF-KB can prolong UPEC’s colonization of the bladder [21]. SAA1 can prevent UPEC’s invasion of urothelial cells and the formation of biofilms [25]. CXCL1 and CXCL10 are also involved in the immune inflammatory response in UTIs [26,27].

The KEGG pathway enrichment analysis suggested the TNF-α signaling pathway is likely the most significant pathway. TNF-α, IL-6, and IL-1β in the TNF-α signaling pathway and CXCL8 were identified as hub genes with the highest enrichment scores. Expression data from the mouse model after UPEC infection showed significant upregulation in expression of the hub genes TNF-α, IL-6, IL-1β and KC in the kidneys of the model group compared to that of the normal group.

In summary, our study used two microarray datasets (GSE43790 and GSE124917) to investigate the differences in expression of inflammatory response-related mRNAs in UTI. We identified a total of 187 DEGs and 20 hub genes, which may be potential targets for the diagnosis and treatment of UTIs. Analyses using the GO and KEGG databases indicate that inflammation, cytokine receptor binding, and TNF-α signaling pathways may be potential targets for the treatment of UTIs.

## Conclusion

In summary, we have constructed and verified pathways and key biomarkers related to urinary tract infections, these results may facilitate the development of new diagnosis and treatment strategies for urinary tract infections. However, additional verification is still needed in the future to explore the role of these biomarkers and pathways.

## Data Availability

The datesets analyzed were acquired from the National Center for Biotechnology Information GEO databases (http://www.ncbi.nlm.nih.gov/geo/).

## Highlights

(1) TNF-α, IL-1β, and IL-6 may be important targets for the diagnosis and treatment of urinary tract infections; (2) The expression of TNF-α, IL-1β, and IL-6 has been verified in mice; (3) Urinary tract infection is closely associated with the TNF signaling pathway.
